# Supplementary material for: A high-resolution mRNA expression time course of embryonic development in zebrafish
Source: eLife. 2017 Nov 16;6:e30860. doi: 10.7554/eLife.30860 (PMC5690287; doi:10.7554/eLife.30860)
Supplement: Supplementary file 6. [file elife-30860-supp6.zip › biolayout-clusters-files/Cluster174-genes.html]

Cluster174


# Cluster174: Genes

| Ensembl ID | Gene Name | Chr | Start | End | Biotype |
| --- | --- | --- | --- | --- | --- |
| ENSDARG00000078442 | NOP9 | 2 | 58418610 | 58446084 | protein\_coding |
| ENSDARG00000041944 | adat2 | 20 | 37830302 | 37857602 | protein\_coding |
| ENSDARG00000011196 | dnajc11a | 23 | 30781306 | 30798378 | protein\_coding |
| ENSDARG00000037109 | pwp2h | 1 | 28095113 | 28146534 | protein\_coding |
| ENSDARG00000075323 | srfbp1 | 19 | 29708968 | 29716565 | protein\_coding |
| ENSDARG00000019293 | tfdp1a | 9 | 35141757 | 35159244 | protein\_coding |
| ENSDARG00000010978 | trmt1 | 11 | 30972941 | 30995296 | protein\_coding |
| ENSDARG00000014692 | znf622 | 2 | 41713733 | 41722114 | protein\_coding |
